# A prospective case control study of functional outcomes and related quality of life after colectomy for neoplasia

**DOI:** 10.1007/s00384-016-2714-3

**Published:** 2016-12-28

**Authors:** Adela Brigic, Samia Sakuma, Richard E. Lovegrove, Paul Bassett, Omar Faiz, Susan K. Clark, Neil Mortensen, Robin H. Kennedy

**Affiliations:** 1 Department of Surgery, St. Mark’s Hospital, Watford Road, Harrow, HA1 3UJ UK; 2 Division of Surgery and Cancer, Imperial College London, London, SW7 2AZ UK; 3 Research and Development Department, North West London Hospitals Trust, Watford Road, London, HA1 3UJ UK; 4 Department of Surgery, Oxford Radcliffe Hospitals, Headley Way, Oxford, OX3 9DU UK; 5 Department of Surgery and Cancer, St. Mary’s Hospital London, W2 1NY, London, UK

**Keywords:** Bowel function, Colorectal neoplasia, Quality of life, MSKCC questionnaire

## Abstract

**Aim:**

Our aim was to assess bowel function and its effect on overall quality of life (QOL) when compared to healthy controls after colectomy.

**Methods:**

Patients undergoing resection of colorectal neoplasia were recruited pre-operatively and followed up at 6 and 12 months, to assess ‘early’ bowel function. Patients who underwent surgery 2 to 4 years previously were recruited for assessment of ‘intermediate’ bowel function. Healthy relatives were recruited as controls. The Memorial Sloan-Kettering Cancer Centre and EQ-5D questionnaires were used to assess bowel function and QOL, respectively. Statistical assessment included regression analyses, parametric and non-parametric tests. The association between QOL and Memorial Sloan-Kettering Cancer Centre (MSKCC) scores was evaluated using Spearman’s rank correlation.

**Results:**

Ninety-one patients were recruited for assessment of ‘early’ and 85 for ‘intermediate’ bowel function. There were 85 controls. Patients had a significantly higher number of bowel movements at each follow-up (*p* < 0.001). At 12 months after surgery, patients reported difficulty with gas-stool discrimination. The ‘intermediate’ group were found to have lower scores for flatus control (<0.001) and total frequency score (*p* 0.03), indicating worse function. Patients with higher total MSKCC scores, no symptoms of urgency and those able to control flatus reported better QOL (*p* 0.006, 0.007 and 0.005, respectively) at 6 and 12 months. Gas-stool differentiation and complete evacuation correlated with better QOL in the ‘intermediate’ bowel function group (*p* 0.02 and 0.02, respectively).

**Conclusion:**

Colonic resection adversely affects elements of bowel function up to 4 years after surgery. Good colonic function, represented by higher MSKCC scores, correlates with better QOL.

## Introduction

Most healthy individuals average one bowel movement per day [1]. Colorectal physiology involves absorption of water and electrolytes, coordinated propulsion of faecal mass from the right colon to the rectum, storage and ultimately, expulsion [2]. The alteration of bowel anatomy after colonic resection can lead to a number of functional disturbances which may be of long-term importance to the patient. Although several studies have reported bowel, urinary and sexual dysfunction after rectal cancer treatment [3–5], the relationship between bowel function and quality of life (QOL) after colonic resection is scarcely reported in the literature.

A few studies have reported an increase in stool frequency following left-sided resection (sigmoid and anterior resection) for the treatment of colorectal cancer [6–9] and diverticular disease [10, 11]. Validated questionnaires to assess bowel function and related QOL were not utilised in these studies. Instead, bowel function is usually reported as numeric data from self-constructed questionnaire surveys [6–9]. This makes it challenging to translate the scanty data that are available into clinically meaningful information for patients. To address the gap in our knowledge, we designed a prospective study to assess ‘early’ (≤12 months after surgery) and ‘intermediate’ (2 to 4 years after surgery) bowel function in patients undergoing hemicolectomy with *en bloc* mesenteric resection (open and laparoscopic). Bowel function and its effect on the QOL were compared to healthy controls. Our proposed hypothesis was that potentially curative hemicolectomy for colonic neoplasia (benign or malignant) adversely affects bowel function and QOL.

## Methods

### Recruitment

To assess the effects of surgery on ‘early’ bowel function, patients were identified during colorectal cancer multidisciplinary meetings in four centres and recruited during their regular pre-operative visits. Patients diagnosed with benign complex colonic polyps [12, 13] unsuitable for endoscopic therapy were included and identified through a specialist multidisciplinary meeting in a single centre. The assessment of ‘intermediate’ bowel function was in eligible patients who had colonic neoplasia resected 2 to 4 years previously. These potential participants were identified from prospectively maintained colorectal cancer databases in two of the hospitals. They were recruited either during their regular follow-up visits or via postal recruitment with a telephone interview. The control group consisted of siblings, partners and spouses recruited for evaluation of ‘early’ bowel function. More than one member of each family was approached to compensate for subjects with no controls.

Inclusion criteria were patients who were either due to undergo, or had undergone, elective right hemicolectomy, transverse colectomy, left hemicolectomy and sigmoid colectomy/high anterior resection (for lesions located above 15 cm from the anal verge); pre-operative ASA grade I, II or III and IUCC stages I–III. Exclusion criteria were inability to consent, diagnosis of inflammatory bowel or coeliac disease, rectal neoplasm, previous pelvic radiation, previous bowel resection (colon, stomach or small bowel), bypass surgery or vagotomy, emergency colonic resection, previous stoma, localised recurrence of disease during the study period and diagnosis of anal incontinence prior to surgery. The same exclusion criteria were applied to the control group.

### Data collection

Sociodemographic and clinical data were recorded for both patient groups. Particular care was taken to record the use of laxatives, opiate-based analgesia or antibiotics within 4 weeks of recruitment for all three groups and at 6 and 12 months after surgery for patients who had been recruited pre-operatively. The caecum, ascending and transverse colon were defined as the ‘right colon’ and descending and sigmoid colon as the ‘left colon’ for comparisons between these subgroups. The length of all resected colonic specimens was recorded after fixation. Post-operative complications with a potential to have an adverse effect on post-operative bowel function including anastomotic leakage, intra-abdominal abscess formation and further abdominal surgery were recorded.

### Study questionnaires

#### MSKCC bowel function questionnaire

Study participants were provided with the Memorial Sloan-Kettering Cancer Centre (MSKCC) bowel function questionnaire designed to evaluate bowel function following rectal resection [14] as we were unable to identify a validated questionnaire developed specifically to evaluate bowel function following colonic resection. The MSKCC questionnaire was designed to measure bowel function rather than quality of life [14, 15] and has been validated against the Faecal Incontinence Quality of Life Scale (FIQL) and the European Organisation of Research and Treatment of Cancer Quality of Life (EORTC) questionnaires: QLQ-C30 (core cancer module) and QLC-38 (colorectal cancer specific module) [14].

The questionnaire consists of 18 items that are grouped into three subscales: frequency, diet and urgency/soilage. The frequency subscale includes six questions regarding the number of bowel movements per 24 h, stool consistency and the ability to get to the toilet on time. The dietary subscale comprises four questions relating to the impact of certain food/drink items on bowel movements and avoidance of those items. The urgency/soilage subscale consists of four questions concerning faecal leakage (day or night and use of pads) and the impact of bowel function on social activities. In addition to the three subscales, four individual questions (Q) of clinical significance are also included: incomplete emptying after a bowel movement [Q4], having a second bowel movement within 15 min [Q6], knowing the difference between gas and bowel movement [Q7] and the ability to control the passage of wind [Q12]. The responses are given on a five-point Likert scale for all items apart from the item asking for the number of bowel movement per 24 h (categorised into quintiles). Within the three subscales, scores for diet and urgency/soilage range from 4 to 20 and frequency from 6 to 30. One total score is obtained by summing all 18 items ranging between 18 and 90. Higher scores indicate better function.

#### EQ-5D quality of life questionnaire

The EuroQol EQ-5D questionnaire measures QOL on five dimensions (mobility, self-care, usual activities, pain/discomfort and anxiety/depression) and has a scale ranging from 0 (no quality of life) to 100 (optimal quality of life). The questionnaire also contains a visual analogue scale (EQ-VAS) (ranging 0 to 100), representing the patient perspective.

### Assessment and follow-up

Patients in the ‘early’ bowel function group were asked to complete questionnaires at recruitment and at 6 and 12 months after surgery. Patients who described a change in bowel habit prior to diagnosis of the colonic neoplasm were asked to report their bowel habit and QOL at the time of recruitment and preceding the onset of bowel symptoms (historic data). Comparison of questionnaire variables between patients and controls was made at baseline and then and six and 12 months follow-up. For the subgroup of patients who reported a change in bowel habit, the ‘historic’ data were used as the baseline values rather than the values obtained immediately prior to surgery.

Both controls and those recruited for assessment of ‘intermediate’ bowel function completed a single set of questionnaires as an assumption was made that a one-off measurement of bowel function and QOL was representative.

### Ethical approval

Ethical approval to conduct the study was obtained from the National Research Ethics Service (NRES) Committee London–Stanmore in May 2011 with a substantial amendment to allow postal follow-up in September 2011 (REC reference 11/LO/0294).

### Power calculation and statistical analysis

The power calculation was based on results from a study conducted by the authors of the MSKCC questionnaire. That study was designed to measure functional outcomes and QOL in patients undergoing rectal cancer surgery [16]. Six-month follow-up data suggested that bowel function was clinically worse when a drop of five points in the median total score was observed [16]. We calculated that a sample size of 85 participants in each group would give 90% power to demonstrate a difference in the median total score of five between the patients and controls, assuming the *α* value of 0.05. To allow for a 10 to 15% loss of patients, we aimed to recruit 98 patients in the ‘early’ bowel function group.

The Fisher’s exact test and the unpaired *t* test were used for analysis of categorical and continuous variables, respectively. Continuous data with skewed distribution were examined using the Mann-Whitney test. Linear regression allowed adjustment for the differences in demographics between the two groups. Log transformation was performed for positively skewed data using linear regression, whereas bootstrapping methods alongside the regression methods were used to analyse negatively skewed outcomes. The results are presented as regression coefficients, which is the mean difference in the outcome between the groups reported as the value for the patient group minus the value for the control group. Therefore, a positive value suggests higher scores and better function for the patient group. The exception is the number of bowel movements per 24 h, which is reported as the ratio of the number of bowel movements in the patient group compared to that observed in the control group. The Spearman’s rank correlation was performed to evaluate the relationship between the bowel function and QOL, as well as between the MSKCC items and the bowel length excised.

## Results

### Demographic data

For assessment of ‘early’ bowel function, a total of 121 patients were recruited prior to surgery and data for 91 patients were analysed. Reasons for exclusion were loss to follow-up (*n* = 15), stoma formation (*n* = 8), death during the study period (*n* = 5), emergency surgery (*n* = 1) and rectal cancer (*n* = 1). Approximately 15% of patients approached in the clinic declined to take part. Eighty-five controls agreed to participate in the study.

A total of 106 patients who underwent surgery 2 to 4 years previously were invited to take part, of whom 85 agreed to participate. All patients approached in the clinics (*n* = 48) agreed to participate with the exception of one. Fifty-eight invitation letters were posted to patients, of whom 40 agreed to participate (71% response rate). Of the non-participants, two were lost to follow-up, six declined to participate and ten did not respond.

Group comparison of demographic data is presented in Table 1. The control group were found to be younger, more likely to be married and to be working at recruitment and less likely to be diabetic, compared to both patient groups. Patients were more likely to use laxatives and anti-diarrhoeal agents 6 months after surgery although this difference was not observed at 12 months. General demographics of patients recruited for assessment of ‘intermediate’ bowel function were similar to that of the patients recruited prior to surgery. This group were also more likely to use laxatives.

**Table 1:** Baseline demographics

	Control group (*n* = 85)	‘Early’ bowel function group^a^		‘Intermediate’ bowel function group^b^	
		(*n* = 91)	*p*	(*n* = 85)	*p*
M:F	34:51	43:47	0.36	49:36	*0.03*
Mean (SD) age	58.2 (13.4)	71.2 (10.5)	*<0.001* ^c^	69.0 (11.2)	*<0.001* ^c^
Ethnicity			0.34		0.07
White	77 (92%)	79 (87%)		68 (81%)	
Other	7 (8%)	11 (13%)		16 (19%)	
Social status					
Single	11 (13%)	5 (5%)	*0.001*	13 (16%)	*0.007*
Married	66 (78%)	64 (70%)		52 (63%)	
Living with partner	7 (8%)	6 (7%)		6 (7%)	
Widowed	1 (1%)	16 (18%)		12 (14%)	
Employment					
Retired	37 (44%)	70 (77%)	*<0.001*	63 (75%)	*<0.001*
Unemployed	5 (6%)	8 (9%)		5 (6%)	
Working	50 (51%)	13 (14%)		16 (19%)	
Laxatives					
Baseline	1 (1%)	8 (9%)	*0.02*	9 (11%)	*0.02*
6 months	1 (1%)	12 (14%)	*0.002*		
12 months	1 (1%)	5 (6%)	0.21		
Antibiotics	1 (1%)	0 (0%)	0.48	5 (6%)	0.21
Anti-diarrhoeal medication					
Baseline	1 (1%)	0 (0%)	0.48	5 (6%)	0.21
6 months	1 (1%)	11 (13%)	*0.005*		
12 months	1 (1%)	5 (6%)	0.21		
Opiates					
Baseline	5 (6%)	2 (2%)	0.27	4 (5%)	1.00
6 months	5 (6%)	0 (0%)	0.06		
12 months	5 (6%)	1 (1%)	0.11		
Diabetes	1 (1%)	10 (11%)	*0.01*	11 (13%)	*0.005*
BMI	27.2 (4.8)	26.1 (5.6)	0.18^c^	27.3 (25.0, 31.4)	0.22^d^

^a^ *p* values correspond to comparison the ‘early’ bowel function group vs. control group

^b^ *p* values correspond to comparison the ‘intermediate’ bowel function group vs. control group

^c^Unpaired *t* test, data presented as mean (SD)

^d^Mann-Whitney test, data presented as median (IQR)

### MSKCC questionnaire

**Fig. 1:**
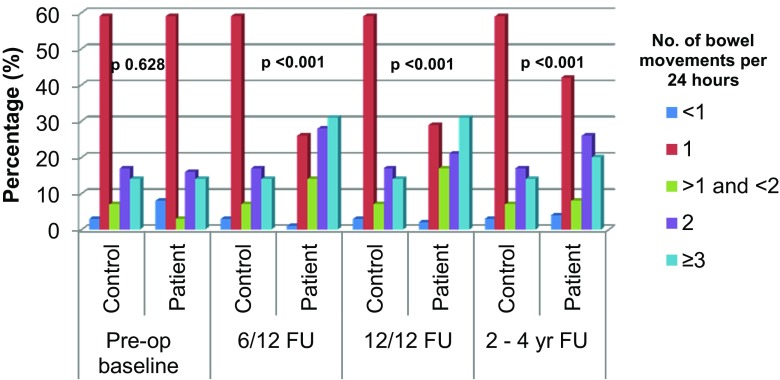
Number of bowel movements split into quintiles at each time point

Thirty-five patients in the group recruited for assessment of ‘early’ bowel function reported a change in bowel habit prior to diagnosis of colonic neoplasia. Therefore, their documented scores preceding the onset of symptoms were used as baseline. For the purposes of this study, we present the number of bowel movements per 24 h independently and include the values in the MSKCC frequency score as per the questionnaire guidelines. Patients experienced a significantly higher number of bowel movements per 24 h (Fig. 1). At 6 months after surgery, 31% of patients experienced more than three motions per day, with an additional 42% experiencing more than one. This compares with figures for controls of 14 and 24%, respectively (*p* < 0.001). At 12 months after surgery, the corresponding figures are 30 and 38% (*p* < 0.001). Patients at 2 to 4 years after surgery reported three or more motions per day in 20% and more than one per day in 34% (*p* > 0.001).

After surgery, there was a trend to lower scores, indicating worse function, in all components of the MSKCC questionnaire except the dietary subclass and Q4 at 12 months after surgery (Table 2). Lower scores were reported in the unadjusted analyses for the urgency item (*p* 0.008) and gas-stool differentiation at six and 12 months after surgery (*p* 0.05 and <0.001, respectively). These changes were also present in the ‘intermediate group’, at 2 to 4 years after surgery, along with difficulty controlling flatus (*p* < 0.001). After adjusting for the differences in demographics, however, statistically significant differences remained for fewer items (Tables 3 and 4). These included an increase in the number of bowel movements at all follow-up points, reduced gas-stool differentiation at 12 months, reduced flatus control and a lower frequency score (worse function) at 2 to 4 years. No association was observed between the bowel length excised and any of the MSKCC items.

**Table 2:** MSKCC questionnaire linear regression analysis for ‘early’ bowel function group

	Median (IQR) score for patient group	Median (IQR) score for control group	*p* ^a^
Baseline early group (*n* = 91)			
Bowel movements per 24 hours	1 (1, 2)	1 (1, 2)	0.40
Frequency score	26 (22, 28)	26 (23, 27)	0.45
Dietary score	19 (16, 20)	16 (14, 18)	*<0.001*
Urgency score	20 (20, 20)	20 (20, 20)	0.87
Complete emptying (Q4)	4 (3, 5)	4 (4, 4)	0.90
2nd bowel movement < 15 min (Q6)	5 (4, 5)	4 (4, 5)	0.09
Gas-stool differentiation (Q7)	5 (4, 5)	5 (5, 5)	0.16
Flatus control (Q12)	4 (4, 5)	4 (4, 5)	0.93
Total score	80 (76, 85)	79 (74, 83)	0.09
6 months follow-up			
Bowel movements per 24 hours	2 (1, 3)	1 (1, 2)	*<0.001*
Frequency score	25 (22, 26)	26 (23, 27)	0.06
Dietary score	18 (15, 20)	16 (14, 18)	*0.01*
Urgency score	20 (18, 20)	20 (20, 20)	*0.008*
Complete emptying (Q4)	4 (3, 4)	4 (4, 4)	0.12
2nd bowel movement < 15 min (Q6)	4 (3, 5)	4 (4, 5)	1.00
Gas-stool differentiation (Q7)	5 (4, 5)	5 (5, 5)	*0.05*
Flatus control (Q12)	4 (4, 5)	4 (4, 5)	0.27
Total score	78 (72, 82)	79 (74, 83)	0.32
12 months follow-up			
Bowel movements per 24 hours	2 (1, 2.5)	1 (1, 2)	*<0.001*
Frequency score	24 (22, 27)	26 (23, 27)	0.07
Dietary score	18 (16, 20)	16 (14, 18)	*0.005*
Urgency score	20 (18, 20)	20 (20, 20)	*0.004*
Complete emptying (Q4)	4 (3, 4)	4 (4, 4)	0.65
2nd bowel movement < 15 min (Q6)	4 (3, 5)	4 (4, 5)	0.67
Gas-stool differentiation (Q7)	5 (4, 5)	5 (5, 5)	*<0.001*
Flatus control (Q12)	4 (4, 5)	4 (4, 5)	0.43
Total score	79 (70, 84)	79 (74, 83)	0.33
Intermediate group (*n* = 85)			
Bowel movements per 24 hours	2 (1, 3)	1 (1, 2)	*<0.001*
Frequency score	24 (21, 26)	26 (23, 27)	*0.03*
Dietary score	18 (16, 20)	16 (14, 18)	*0.009*
Urgency score	20 (19, 20)	20 (20, 20)	*0.04*
Complete emptying (Q4)	4 (3, 5)	4 (4, 4)	0.10
2nd bowel movement < 15 min (Q6)	4 (3, 5)	4 (4, 5)	0.95
Gas-stool differentiation (Q7)	5 (4, 5)	5 (5, 5)	*0.002*
Flatus control (Q12)	4 (3, 4)	4 (4, 5)	*<0.001*
Total score	81 (73, 84)	79 (74, 83)	0.08

^a^Mann-Whitney test

**Table 3:** MSKCC questionnaire linear regression analysis for ‘early’ bowel function group

	Mean (95% CI) group difference^a^	*p*
Baseline ‘early’ bowel function group [*n* = 91]^b^		
Bowel movements per 24 hours^c^	0.95 (0.78, 1.16)	0.61
Frequency score	0.1 (−1.2, 1.4)	0.92
Dietary score	1.1 (0.2, 2.1)	*0.02*
Urgency score	0.3 (−0.3, 1.0)	0.39
Complete emptying (Q4)	0.1 (−0.3, 0.5)	0.75
2nd bowel movement < 15 min (Q6)	0.2 (−0.1, 0.6)	0.26
Gas-stool differentiation (Q7)	0.0 (−0.3, 0.2)	0.75
Flatus control (Q12)	−0.1 (−0.4, 0.2)	0.72
Total score	1.8 (−1.2, 4.2)	0.19
‘Early’ bowel function group at 6 months^d^		
Bowel movements per 24 hours^c^	1.59 (1.31, 1.92)	*<0.001*
Frequency score	−0.3 (−1.6, 0.9)	0.61
Dietary score	0.1 (−0.9, 1.2)	0.90
Urgency score	−0.6 (−1.5, 0.2)	0.15
Complete emptying (Q4)	−0.1 (−0.5, 0.3)	0.52
2nd bowel movement < 15 min (Q6)	−0.1 (−0.6, 0.3)	0.48
Gas-stool differentiation (Q7)	−0.1 (−0.4, 0.2)	0.31
Flatus control (Q12)	−0.1 (−0.4, 0.2)	0.55
Total score	−1.1 (−4.1, 2.1)	0.51
‘Early’ bowel function group at 12 months^e^		
Bowel movements per 24 hours^c^	1.45 (1.21, 1.75)	*<0.001*
Frequency score	−1.2 (−2.7, 0.1)	0.07
Dietary score	0.7 (−0.3, 1.6)	0.16
Urgency score	−0.5 (−1.4, 0.3)	0.24
Complete emptying (Q4)	0.1 (−0.3, 0.4)	0.72
2nd bowel movement < 15 min (Q6)	−0.1 (−0.6, 0.3)	0.54
Gas-stool differentiation (Q7)	−0.4 (−0.7, −0.1)	*0.01*
Flatus control (Q12)	−0.4 (−0.8, 0.0)	0.06
Total score	−1.5 (−4.6, 1.6)	0.36

^a^Regression coefficient where a higher value indicates higher score and therefore better function for patients

^b^Adjusted for age, marital status, employment, laxatives at baseline and diabetes

^c^Due to log transformation, results are reported as the ratio of number of movements in the patient group

^d^Adjusted for age, marital status, employment, diabetes, laxatives and anti-diarrhoeal medication

^e^Adjusted for age, marital status, employment and diabetes

**Table 4:** MSKCC questionnaire linear regression analysis for ‘intermediate’ bowel function group

	Mean (95% CI) group difference^a^	*p*
‘Intermediate’ bowel function group [*n* = 85]^b^		
Bowel movements per 24 hours^c^	1.44 (1.20, 1.73)	*<0.001*
Frequency score	−1.5 (−3.0, 0.0)	*0.03*
Dietary score	0.6 (−0.4, 1.6)	0.23
Urgency score	−0.6 (−1.5, 0.3)	0.20
Complete emptying (Q4)	−0.1 (−0.5, 0.3)	0.47
2nd bowel movement < 15 min (Q6)	−0.1 (−0.5, 0.3)	0.54
Gas-stool differentiation (Q7)	−0.2 (−0.5, 0.1)	0.12
Flatus control (Q12)	−0.6 (−1.0, −0.3)	*<0.001*
Total score	−2.7 (−36.0, 0.7)	0.11

^a^Regression coefficient where a higher value indicates higher score and therefore better function for patients

^b^Adjusted for age, sex, marital status, employment, laxative use and diabetes

^c^Due to log transformation, results are reported as the ratio of number of movements in the patient group

### EQ-5D questionnaire

No difference in the QOL between patients and controls was observed at any time point (Table 5). A weakly positive correlation was observed between the EQ-VAS score and flatus control (rs 0.30, *p* 0.005), the urgency score (rs 0.29, *p* 0.007) and the total MSKCC score (rs 0.31, *p* 0.006) at 6 months after surgery, indicating that those with higher scores have better QOL. A similar relationship between the EQ-VAS and complete emptying was seen at 12 months (rs 0.34, *p* 0.01). In the ‘intermediate’ bowel function group, complete evacuation was positively correlated with both EQ-5D-QOL and EQ-VAS (rs 0.29, *p* 0.007 and rs 0.25, *p* 0.02, respectively) whereas gas-stool differentiation was correlated with EQ-5D-QOL variable only (*p* 0.02).

**Table 5:** EQ-5D questionnaire linear regression analysis

	Mean (95% CI) group difference^a^	*p*
Baseline ‘early’ bowel function group [*n* = 91]^b^		
EQ-5D-QOL	−0.01 (−0.07, 0.05)	0.78
EQ-VAS	−2.5 (−9.3, −3.9)	0.45
‘Early’ bowel function group at 6 months^c^		
EQ-5D-QOL	−0.02 (−0.08, 0.04)	0.52
EQ-VAS	−3.9 (−9.6, 1.8)	0.18
‘Early’ bowel function group at 12 months^d^		
EQ-5D-QOL	0.00 (−0.06, 0.06)	0.95
EQ-VAS	−0.3 (−5.9, 6.1)	0.93
‘Intermediate’ bowel function group [*n* = 85]^e^		
EQ-5D-QOL	−0.04 (−0.11, 0.04)	0.31
EQ-VAS	−2.1 (−7.8, 3.6)	0.47

^a^Regression coefficient where a higher value indicates higher score and therefore better function for patients

^b^Adjusted for age, marital status, employment, diabetes and laxatives

^c^Adjusted for age, marital status, employment, diabetes, laxatives and anti-diarrhoeal medication

^d^Adjusted for age, marital status, employment and diabetes

^e^Adjusted for age, sex, marital status, employment, laxative use and diabetes

### Right- vs. left-sided resections

Both patient cohorts undergoing right- or left-sided resections had similar demographic details and post-operative outcomes (Tables 6 and 7). Care involved the use of an enhanced recovery programme in all patients and over 80% of procedures were laparoscopic. Anastomotic leakage occurred only in two patients, recruited for the assessment of ‘early’ bowel function group. These followed left-sided resections and both were successfully treated with antibiotics only.

**Table 6:** Demographics details of right- vs. left-sided colonic resections for patients recruited for assessment of ‘early’ bowel function

	Right-sided resection (*n* = 47)	Left-sided resection (*n* = 44)	*p*
M:F	22 (47%):25 (53%)	21 (49%):22 (51%)	1.00
ASA grade			0.22
I	7 (18%)	9 (26%)	
II	20 (51%)	21 (60%)	
III	12 (31%)	5 (14%)	
Type of surgery			0.09
Laparoscopic	41 (87%)	37 (86%)	
Open	5 (11%)	1 (2%)	
Converted	1 (2%)	5 (12%)	
Median (IQR) LOS, days^a^	6 (4, 10)	5 (4, 7)	0.29
30-day complications	14 (30%)	13 (30%)	1.00
30-day readmission	0 (0%)	5 (12%)	*0.02*
30-day reoperation	0 (0%)	0 (0%)	–
Mean (SD) specimen length cm^b^	29.4 (12.8)	27.2 (9.1)	0.36
IUCC stage			0.19
I	9 (20%)	11 (26%)	
II	18 (41%)	14 (33%)	
III	17 (39%)	14 (33%)	
Benign	0 (0%)	4 (9%)	
Distant recurrence at 6 months	2 (5%)	0 (0%)	0.50
Distant recurrence at 12 months	2 (4%)	1 (2%)	1.00
Chemotherapy	19 (40%)	16 (34%)	0.97

^a^Mann-Whitney test

^b^Unpaired *t* test

**Table 7:** Demographic details of right- vs. left-sided colonic resections for patients recruited for assessment of ‘intermediate’ bowel function

	Right-sided resections (*n* = 48)	Left-sided resections (*n* = 37)	*p*
M:F	26 (54%):22 (46%)	10 (27%):27 (73%)	0.015
ASA grade			0.22
I	7 (15%)	8 (22%)	
II	33 (68%)	26 (70%)	
III	7 (15%)	1 (3%)	
Not available	1 (2%)	2 (5%)	
Type of surgery			0.25
Laparoscopic	38 (79%)	32 (87%)	
Open	8 (16%)	3 (8%)	
Converted	2 (5%)	2 (5%)	
Median (IQR) LOS, days^a^	4 (3, 6)	5 (4, 6)	0.15
30-day complications	14 (29%)	6 (16%)	*0.02*
30-day readmission	4 (8%)	1 (3%)	0.30
30-day reoperation	1 (2%)	1 (3%)	0.68
Mean (SD) specimen length cm**	42.1 (17.4)	28.4 (10.3)	*<0.001*
IUCC stage			0.19
I	6 (12%)	10 (27%)	
II	23 (48%)	12 (32%)	
III	19 (40%)	15 (41%)	
Distant recurrence	1 (2%)	2 (5%)	0.50

^a^Mann-Whitney test

** unpired *t* test

No difference in QOL between the groups was observed at any time point after surgery. At 6 and 12 months follow-up, patients who underwent left-sided resections experienced a higher number of bowel movements [2.0 (1.5, 3.3) vs. 1.5 (1.0, 2.5), *p* 0.04 and 2.0 (1.5, 3.0) vs. 1.5 (1.0, 2.0), *p* 0.002, respectively]. They also reported lower scores for Q6 (needing a second bowel movement within 15 min) indicating worse outcome [4 (3, 5) vs. 5 (4, 5) at both time points, *p* 0.001 and 0.01, respectively]. Although there was no statistically significant difference between the groups for the number of bowel movements per 24 h in the ‘intermediate group’, overall, a lower frequency score was observed following right-sided resections [24 (21, 26) vs. 25 (23, 27), *p* 0.03], indicating worse function.

## Discussion

Almost 50% of patients undergoing surgery for colonic neoplasia have concerns about post-operative bowel function [17]. This study was motivated by the need to generate an estimate of bowel dysfunction after colectomy and its effect on QOL, as the literature on the subject is sparce. Assuming that the total MSKCC score represents the overall bowel function, the results of our study suggest that bowel function and QOL after colectomy for neoplasia are comparable to that of healthy controls. However, if one considers bowel frequency alone, it is significantly increased in one third of patients at 6 and 12 months after surgery and in one quarter at 2 to 4 years. One in five patients has more than three motions per day 2 or more years after surgery. At 1 year after surgery, patients reported having difficulty with gas-stool discrimination and at 2 to 4 years, there is difficulty controlling flatus. However, patients do not seem to perceive problems identified after surgery as affecting their overall QOL when assessed by EQ-5D and EQ-VAS scores. Despite this, correlation existed between several items of the MSKCC and better QOL: lack of urgency, an ability to control flatus, full evacuation and satisfactory gas-stool differentiation.

This is the largest study published to date designed to evaluate bowel function following colectomy for neoplasia. Its strengths include prospective design, a large number of patients, inclusion of controls and assessment of early and intermediate outcomes. We utilised a validated questionnaire designed specifically to assess bowel function following rectal resections. Although such a questionnaire is not currently available for patients following colectomy, the results of this study suggest that some patients experience bowel dysfunction and therefore could be used as a platform for future studies of qualitative design. We also utilised a well-established QOL questionnaire that is simple and easy to complete, choosing this to maximise compliance.

The observed increase in bowel frequency after surgery in a proportion of our patients could be due to a number of factors. It has been proposed that high ligation of the inferior mesenteric artery at left hemicolectomy can lead to damage of the lower mesenteric ganglion [18–20], increased colonic motility and diarrhoea [21]. Loss of the ileocaecal valve [22] and excision of the right colon [23] may lead to bile acid malabsorption, increased motility, mucus secretion and stimulation of defecation [24]. The right colon has the greatest colonic absorptive capacity [25] and the contents reside there for longer than other parts, which may explain the observed increased incidence of diarrhoea following right hemicolectomy. Adachi et al. [7] reported that increased specimen length was associated with worse functional outcomes following sigmoid colectomy (*p* < 0.05). We did not find this: however, the number of patients included in this study was relatively small and colonic specimens were measured after fixation in formalin, making conclusions difficult to translate to clinical practice.

Although we found a weak correlation between several MSKCC items and the EQ-5D QOL and EQ-VAS scores, the overall QOL of patients in our study at each follow-up point was comparable to controls, in keeping with the previously published studies. Theodoropoulus et al. [26] reported the use of more detailed QOL questionnaires including SF-36, EORTC QLQ-C30, QLQ-CR29 and GIQLI questionnaire to evaluate patients’ QOL at 1, 6 and 12 months after laparoscopic colectomy. At 6 and 12 months after surgery, almost all QOL scores were better than baseline and were comparable to the general population values. In addition, Ramsey et al. [5] reviewed QOL of long-term CRC survivors more than 5 years after their initial treatment and found that patients had a relatively high perceived QOL compared to age-matched controls. It may be that the use of a more complex, and presumably sensitive, QOL measure would have identified a reduction in early QOL but we chose to use a relatively simple approach in order to encourage recruitment and avoid loss to follow-up.

Despite the prospective nature of this adequately powered case-controlled study and the use of validated questionnaires, there are several potential limitations that may affect the generalisability of our conclusions. A selection bias may exist as only patients and controls willing and able to take part in the study were recruited. Recall bias is common in self-reported surveys and a proportion of our patients presented with a change in bowel habit which may have had an effect on the baseline values for both the MSKCC and EQ-5D questionnaires. Although loss to follow-up was relatively low (12%), these patients may have been embarrassed to talk about their bowel function after surgery due to the severity of their symptoms, thus potentially minimising functional problems reported after colectomy. In addition, having survived cancer may have strengthened positive health perceptions in this relatively older patient population when they compared their health to that at the time of cancer diagnosis. Our patient population was older than controls and had a higher incidence of diabetes. However, when adjusted for those demographic differences, the MSKCC and EQ5D scores reported by both groups were comparable at baseline.

The results of this study suggest that aspects of bowel function may be adversely affected years after colectomy, and further study would be beneficial in order define the problem and improve treatment. It would be appropriate to inform patients who wish to know that increased bowel frequency after surgery affects approximately one in five. A study with longer follow-up would clarify the discrepancy observed between the ‘early’ and ‘intermediate’ bowel function following different resections, and semi-structured interviews might help identify issues that are specific for this patient group. In addition, the adverse functional outcomes identified in this study may provide an incentive to find an alternative to segmental colectomy for patients who are likely to be node negative [27].
